# Anticoagulation for mechanical heart valves during pregnancy: A case report and a literature review

**DOI:** 10.1097/MD.0000000000032550

**Published:** 2022-12-30

**Authors:** Chunqiang Bai, Haiying Wu, Wenying Wu, Peiming Feng, Minghui Nie, Li Zhao, Fanyue Meng

**Affiliations:** a Department of Ultrasonography, The Affiliated Hospital of Chengde Medical University, Chengde, China; b Department of Obstetrics, The Affiliated Hospital of Chengde Medical University, Chengde, China; c Department of MEC Ultrasound, Chengde Center Hospital, The Second Affiliated to Chengde Medical University, Chengde, China.

**Keywords:** anticoagulation, echocardiography, mechanical heart valves, pregnancy, thromboembolism

## Abstract

Most previous treatment guidelines for pregnant women with mechanical heart valves recommend that low molecular weight heparin (LMWH) should be applied once every 12 hours and only as required to reach peak anti-Xa levels of approximately 1.0 to 1.2 IU/mL, but it is commonly associated with subtherapeutic trough levels, consequently with an inadequate level of anticoagulation. Our case report here together with a literature review suggests that dose-adjusted (Target trough anti-Xa levels of 0.6 to 0.7 IU/mL and with peak anti-Xa levels of around 1.0 to 1.2 IU/mL or < 1.5 IU/mL) LMWH should be given thrice daily throughout pregnancy. In addition, the findings of this rare case indicate that a combination of LMWH and warfarin is effective in the treatment of small thromboses in pregnancy.

**Patient concerns:**

In the 1^st^ trimester of pregnancy, a 28-year old pregnant female with a mechanical valve had a significant increase in the aortic valve flow rate and suspected mechanical valve thrombosis.

**Diagnoses:**

The peak velocity of the pregnant female aortic mechanical valve increased, and mechanical valve thrombosis was suspected.

**Interventions:**

We adjusted the enoxaparin sodium dose every 12 hours to 1 injection every 8 hours, with a total daily dose of 160 mL. Based on the original application of LMWH, warfarin (3 mg/day) was recommended.

**Outcomes:**

The pregnant woman delivered a live baby by cesarean section, and the peak flow velocity of the mechanical valve in the aortic position was reduced to nearly equivalent to the patient’s pre-pregnancy status. The mother and the baby were in good health at the time of discharge.

**Lessons:**

LMWH is administered twice daily, and anti-Xa trough levels are mostly in a subtherapeutic state, which may lead to insufficient anticoagulation and thrombosis. Dose-adjusted LMWH thrice daily throughout pregnancy is the recommended treatment for pregnant women with mechanical heart valves. The combination of LMWH and warfarin exhibited good efficacy for the treatment of small thromboses.

## 1. Introduction

Pregnancy is a complex physiological process involving changes in the mother’s coagulation and fibrinolysis systems, including acquired protein C resistance, reduced levels of protein S, production of procoagulant factors, and reduced levels of fibrinolysis. These changes can create a hypercoagulable state.^[1–3]^ The concurrent incidence of venous stasis and hypercoagulability results in an almost 5-fold increase in the risk of venous thromboembolism during pregnancy.^[4]^ One-third of all cases of heart disease in pregnancy are valvular.^[5]^ Mechanical heart valves (MHVs) can further increase the risk of thromboembolism, thus leading to the need for systemic anticoagulation; at present warfarin is the safest anticoagulant for use in pregnant females with MHVs.^[6,7]^ However, warfarin is a small-molecular-weight compound that can easily cross the placenta^[8,9]^ and may cause embryonic disease (nasal hypoplasia, stippled epiphyses, central nervous system, or ocular abnormalities) or fetal death.^[9]^ This makes the choice of anticoagulation regimen for pregnant women with MHVs challenging. Compared with warfarin, low molecular weight heparin (LMWH) is known to be much safer for the fetus because it cannot cross the placenta.^[10,11]^ However, few studies on unmonitored therapy have questioned whether it is as effective as warfarin for stroke prophylaxis in patients with MHVs.^[12–14]^ Thus, there is no clear consensus regarding the optimal anticoagulation strategy for pregnant women with MHVs. However, in the present case, the entire course of LMWH was administered 3 times daily, with the dose adjusted according to the trough and peak levels of anti-Xa, which exhibited good anticoagulation effects. Consequently, this regimen should be recommended as the first-choice treatment for pregnant women with mechanical valves.^[14,15]^ Most previous treatment guidelines recommend the application of LMWH once every 12 hours to reach peak anti-Xa levels. However, the present case shows that for pregnant women with a mechanical valve, it is preferable to adjust the dose of LMWH every 8 hours to reach peak and trough anti-Xa levels. Measurement of trough levels is particularly important,^[16–18]^ and the combination of LMWH and warfarin is effective in the treatment of small thromboses during pregnancy.

## 2. Case presentation

Our patient was 28 years of age and weighed 52 kg. Owing to rheumatic disease, the patient underwent bileaflet mechanical replacement of the mitral and aortic valves. The peak velocities of the mechanical valve in the aortic and mitral positions were 3.0 m/s and 2.1 m/s respectively, with mean cross valve pressure difference of 22 mm Hg and 7 mm Hg respectively. Warfarin (4 mg) was taken orally every day after surgery and the international normalized ratio (INR) was controlled at between 2 to 3. Thirty days after menopause, blood tests revealed that the patient was positive for human chorionic gonadotropin, and was therefore classified as pregnant. Owing to the patient’s concerns regarding the toxicity of warfarin to the fetus, warfarin was discontinued, and subcutaneous injections (0.6 mL) of enoxaparin sodium LMWH were administered twice daily. During this period, the patient’s weight remained stable. Because local hospitals cannot detect anti-Xa levels, the peak anti-Xa levels cannot be monitored. The peak flow velocities and mean cross-valve pressures of the mechanical valve in the aortic and mitral positions did not change during early pregnancy stages. By the 11^th^ week of pregnancy, echocardiography showed that the peak velocity of the mechanical valve in the aortic position was 3.7 m/s but was unchanged in the mitral position. Therefore, we adjusted the enoxaparin sodium dose to 1 injection every 8 hours, with a total daily dose of 160 mL. By the 13^th^ week of pregnancy, echocardiography showed that the peak velocity of the aortic mechanical valve had increased to 4.3 m/s, with a mean cross-valve pressure difference of 40 mm Hg. Two days later, the peak velocity of the aortic mechanical valve reached a maximum pregnancy-related value of 4.5 m/s, with a mean cross-valve pressure difference of 49 mm Hg. Considering the possibility of aortic mechanical valve thrombosis, the fetus was no longer sensitive to the effects of warfarin during the second and third trimesters. Therefore, based on the original application of LMWH, warfarin (3 mg/day) was recommended. After 4 days of warfarin treatment, the INR reached 2.1, and the peak flow velocity of the aortic mechanical valve began to gradually decrease, finally reaching 3.5 m/s with a mean cross-valve pressure difference of 28 mm Hg at the 15^th^ week of pregnancy. The treatment regimen was continued until labor. During this period, the INR fluctuated between 1.5 to 2.0 and the warfarin dose was adjusted accordingly. The peak and trough levels of anti-Xa were 0.9 U/mL and 0.6 U/mL, respectively. From this point (15 weeks), the patient experienced stable middle and late pregnancies, without chest tightness, palpitations, or any other discomfort. Warfarin was stopped at 36 weeks of gestation.

At 37 weeks of gestation, LMWH was stopped,and 24 hours later, the pregnant woman delivered a live baby by cesarean section, with a neonatal Apgar score of 10-10-10. Six hours later, warfarin (3 mg/day) and enoxaparin 0.6 mg every 12 hours. On the 3rd day after surgery, echocardiography showed that the peak flow velocity of the mechanical valve in the aortic position was reduced to 2.9 m/s, and the mean transvalvular pressure difference was 20 mm Hg, which was nearly equivalent to the patient’s pre-pregnancy status. The mother and the baby were in good health at the time of discharge.

## 3. Discussion

Various anticoagulation strategies have been proposed by different professional groups for pregnant women with MHVs. These strategies include dose-adjusted LMWH twice a day for the first trimester (peak anti-Xa 0.8 to 1.2 U/mL, LMWH 4 hours post dose) followed by warfarin in the second and third trimesters; dose-adjusted (to achieve peak anti-Xa 0.8 to 1.2 U/mL LMWH 4 hours post dose) LMWH twice a day throughout pregnancy; or low-dose warfarin throughout in women on low dose warfarin.^[14]^ The above range of strategies demonstrates the lack of clear consensus on the ideal anticoagulation strategy for pregnant women with MHVs. In this case report, we describe a novel anticoagulation strategy for pregnant women with MHVs.

Although warfarin is the safest anticoagulant for the mother when used during pregnancy, it has the potential to cause fatal disease.^[19]^ However, the true incidence of fetal embryopathy when warfarin is administered remains controversial, and appears to depend on the dose and duration of pregnancy exposure.^[9]^ The sensitive period for teratogenic effects is between the 6^th^ and 12^th^ weeks of pregnancy; therefore, warfarin should be discontinued and replaced with LMWH.^[20]^ Previous studies have reported that the risk of fetal disease is reduced at lower warfarin doses^[21]^; however, other studies have suggested that low doses of warfarin do not alter the risk of fetal disease.^[22–24]^ Published case studies have demonstrated that fetal disease may occur despite administration of low doses of warfarin.^[25–27]^ Considering the adverse effects of warfarin on fetuses. After the diagnosis of pregnancy at 4 weeks, warfarin was immediately discontinued and the regimen was changed to anticoagulation with LMWH. Due to the failure to monitor anti-Xa levels during early pregnancy in this case, the anticoagulation effects of subcutaneous injection (60 mg) of enoxaparin every 12 hours in the first trimester were insufficient, and the peak flow rate and mean cross-valve pressure of the mechanical aortic valve increased gradually. It is possible that the valve was thrombosed. However, the patient refused transesophageal echocardiography for further diagnosis owing to discomfort caused by the esophageal ultrasound, despite clear indications that anticoagulation had become a problem. Adjusting the dosage of LMWH without monitoring the levels of anti-Xa is the most common cause of thrombosis because of the insufficient anticoagulation effects of LMWH.^[14,17,28]^

American College of Cardiology/American Heart Association guidelines require only monitoring of pregnant woman with peak anti-Xa levels between 0.8 to 1.2 U/mL when LMWH is administered. However, optimal LMWH should be administered twice or thrice daily, and anti-Xa level monitoring regimens are yet to be established.^[29]^ Some studies have indicated that both peak and trough anti-Xa levels should be monitored during LMWH administration.^[16]^ Some data also indicate that when LMWH is administered twice a day, anti-Xa trough levels are mostly in a subtherapeutic state.^[17]^ This may have led to insufficient anticoagulation therapy and development of thrombosis. Several studies have shown that despite maintaining therapeutic peak anti-Xa levels of LMWH, 73% to 91% of patients on a twice-daily LMWH regimen exhibited trough anti-Xa levels that were in a sub-therapeutic state.^[15,17,28,30]^ While peak anti-Xa levels are not a reliable guide for LMWH therapy, monitoring and maintenance of trough levels are necessary to ensure adequate anticoagulation in pregnant women with MHVs.^[30]^

Our patient received enoxaparin 3 times a day, and the total dose was increased to 160 mg/day. This 3-time daily regimen is preferred in terms of pharmacokinetics. The characteristics of anticoagulation during pregnancy include increased maternal blood volume, distribution volume, glomerular filtration rate, binding of heparin to proteins, and the removal of heparin by the kidneys. The half-life and peak levels of heparin and LMWH decrease during pregnancy. Therefore, higher doses and more frequent adjustments were needed.^[31]^ Furthermore, the half-life of enoxaparin sodium after a single subcutaneous injection was approximately 4 hours and that after twice-daily administration was approximately 7 hours. As the LMWH interval decreased, the half-life of enoxaparin sodium increased. From this perspective, an 8-hourly dosing is more stable, reducing the risk of the peak concentration of enoxaparin sodium becoming sufficiently high to cause bleeding, which also avoids the risk of thrombosis due to the low trough concentration of enoxaparin sodium. In the present case, when the patient was injected subcutaneously with LMWH once every 8 hours, we found that the peak and trough levels of anti-XA were 0.9 U/mL and 0.6 U/mL, respectively. Consequently, the drug concentration fluctuated only slightly, eventually reaching the therapeutic range. This further confirms the benefits of 8-hourly rather than 12-hourly dosing ^[18]^. Target trough anti-Xa levels of 0.6 to 0.7 IU/mL with peak anti-Xa levels of around 1.0 to 1.2 IU/mL or < 1.5 IU/mL have been recommended ^[18]^, and LMWH thrice daily is better than LMWH twice daily to achieve the Target anti-Xa levels.^[16,18]^

In our patient, transthoracic echocardiography suggested a mechanical thrombosis of the aortic valve. Therefore, at the 13^th^ week of pregnancy and after the sensitive period for warfarin-related teratogenesis had passed,^[10]^ the patient commenced LMWH and warfarin (3 mg), thus creating simultaneous double anticoagulant therapy.^[29,32]^ The effect of warfarin is amplified in fetal blood, resulting in microbleeding, which can lead to fetal death and embryonic diseases.^[9]^ Considering this, we controlled the INR mainly range of 1.8 to 2.0, lower than the recommended range of 2.5 to 3.5,^[25,29,32]^ although a target INR of 1.5 to 2.5 has been suggested for pregnant women with low thrombotic risk.^[25]^ The mechanical valve velocity in the aortic valve position of our patient gradually decreased when INR reached 2, indicating that the combined administration of LMWH and warfarin was effective. Our experience indicates that the combination of LMWH and warfarin is effective for the treatment of thrombosis in pregnant women with mechanical valves, and should be widely applied and recommended. Since thrombolytic therapy and surgical treatment are risky and traumatic for pregnant women and fetuses, they are not considered optimal.^[29]^

Echocardiography plays a vital role in the evaluation of flow velocity to assess the mechanical valve function and anticoagulant efficacy. Physicians should be aware of this role and perform the procedure at least every 2 weeks in pregnant women with MHVs. When patients have relevant symptoms, echocardiography should be performed immediately and the patient should be closely followed up. This is because ultrasound allows for the timely detection of changes in flow velocity and differential pressure in mechanical valves as well as an asymptomatic thrombus.^[29]^

## 4. Conclusion

The impact of warfarin on the fetus cannot be ignored, and the risks associated with its use in treatment should always be minimized. For pregnant women with MHVs, dose-adjusted LMWH (Target trough anti-Xa levels of 0.6 to 0.7 IU/mL with peak anti-Xa levels of around 1.0 to 1.2 IU/mL or < 1.5 IU/mL) thrice daily throughout pregnancy as described above is the recommended choice of treatment at present. The combination of LMWH and warfarin exhibited good efficacy for the treatment of small thromboses. Finally, echocardiographic monitoring is very important for pregnant women with mechanical valves during anticoagulant treatment and should be performed once every 2 weeks.

## Author contributions

**Conceptualization:** Chunqiang Bai, Fanyue Meng, Minghui Nie.

**Data curation:** Wenying Wu, Haiying Wu.

**Formal analysis:** Haiying Wu.

**Investigation:** Peiming Feng.

**Resources:** Li Zhao.

**Writing – original draft:** Chunqiang Bai.

**Writing – review & editing:** Chunqiang Bai.
